# Anti-Inflammatory Effects of *Weigela subsessilis* Callus Extract via Suppression of MAPK and NF-κB Signaling

**DOI:** 10.3390/plants10081635

**Published:** 2021-08-09

**Authors:** Hyeon-Ji Lim, Eun Yee Jie, In-Sun Park, Sang-Jun Kim, Woo Seok Ahn, Seung-Il Jeong, Suk Weon Kim, Chan-Hun Jung

**Affiliations:** 1 Jeonju AgroBio-Materials Institute, Jeonju-si 54810, Jeollabuk-do, Korea; lhj0923@jami.re.kr (H.-J.L.); witwit58@jami.re.kr (I.-S.P.); process95@jami.re.kr (S.-J.K.); sijeong@jami.re.kr (S.-I.J.); 2 Biological Resource Center, Korea Research Institute of Bioscience & Biotechnology, Jeoneup-si 56212, Jeollabuk-do, Korea; jeannie@kribb.re.kr (E.Y.J.); dntjr0412@kribb.re.kr (W.S.A.)

**Keywords:** *Weigela subsessilis*, callus, antioxidant, antibacterial activity, anti-inflammatory, inflammatory disorder

## Abstract

*Weigela subsessilis* is used in folk medicine to treat pain and allergic syndromes in Korea. However, the antibacterial and anti-inflammatory activities of *W. subsessilis* callus extract remain unexplored. In this study, we aimed to evaluate the *W. subsessilis* callus of pharmacological activity. Therefore, we first established in vitro calluses of *W.* *subsessilis* via plant tissue culture methods. We then evaluated the antioxidant and anti-inflammatory effects of *W. subsessilis* callus extract in lipopolysaccharide (LPS)-treated RAW264.7 macrophage cells. The *W. subsessilis* callus extract showed antioxidant and anti-inflammatory effects. These effects were regulated via suppression of mitogen-activated protein kinase signaling through LPS-induced translocation of nuclear factor kappa B (NF-κB) p65 from the cytoplasm to the nucleus. *W. subsessilis* callus extract also showed antibacterial and anti-inflammatory activities in *Propionibacterium acnes*-treated HaCaT keratinocyte cells. These results indicate that *W. subsessilis* callus extract has antioxidant, antibacterial and anti-inflammatory activities, suggesting its possible application in the treatment of inflammatory disorders.

## 1. Introduction

*Weigela subsessilis* L. H. Bailey, a member of the family Caprifoliaceae, is widespread in East Asian countries. *W. subsessilis* (WS) has been cultivated specifically in Korea and Japan. It is used in folk medicine to treat pain and allergic syndromes in Korea. WS has various pharmacological activities, including anti-complement activation effect, stimulation of melanogenesis, inhibition of low-density lipoprotein oxidation, stimulation of glucose uptake and anti-inflammatory activity [1,2,3,4]. Studies on the chemical constituents of WS have shown the presence of flavonoids, coumarins, terpenoids, sterols and iridoids [5]. Among the chemical constituents of WS, coumarins, terpenoids and iridoids have an equally broad spectrum of pharmacological functions, such as anti-cancer, anti-inflammatory and antibacterial activities [6,7,8,9]. Other chemical constituents of WS, including flavonoids and sterols, also exhibit antioxidant activities [10,11,12].

Plant cell and tissue culture methods are efficient and powerful tools for the genetic transformation of useful plant genotypes with high-value recombinant proteins and the continuous production of plant-derived metabolites with commercial value [13,14]. Plant cell or tissue cultures capable of producing useful metabolites offer several advantages over traditional field cultivation [15]. Among them, the biggest advantage is that plant cells can be easily proliferated for mass production under aseptic culture conditions.

Even though WS has various pharmacological activities, to the best of our knowledge, there have been no reports of in vitro proliferation or callus induction through plant tissue culture. Therefore, this study aimed to evaluate whether the WS callus (WSC) extract retains the pharmacological activities of the parent plant after establishing in vitro callus cultures of WS to continuously produce materials of equal quality.

## 2. Results and Discussion

### 2.1. In Vitro Proliferation of W. subsessilis Callus

**Figure 1:**
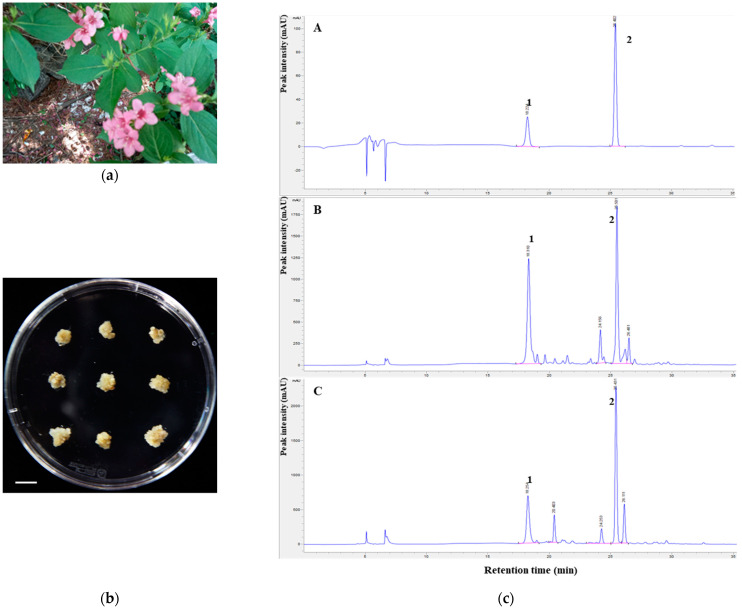
Establishment of an in vitro proliferation system from stem explants of *W. subsessilis*: (**a**) Field grown plant of *W. subsessilis*; (**b**) In vitro grown calluses of *W. subsessilis*. Scale bar, 1 cm; (**c**) HPLC chromatograms of scopolin (1) and scopoletin (2) in extracts of *W. subsessilis*. A methanol extract of *W. subsessilis* callus was analyzed compared to standard peaks with octadecyl-silica analytical column (**A**), standard; (**B**), callus of *W. subsessilis*; (**C**), leaves of *W. subsessilis*).

Calluses were successfully induced from stem explants of WS by culturing on 1/2MS1B03D medium after four weeks of incubation. The calluses were then transferred to the same fresh medium and sub-cultured every four weeks (Figure 1b). Proliferated calluses obtained from the subculture were collected carefully and freeze-dried for the antioxidant, antibacterial and anti-inflammatory assays. Next, to confirm the quality of this plant material, the chemical constituents of WSC and leaves of WS were compared using a high-performance liquid chromatography (HPLC) with diode array detector analysis. As shown in Figure 1c, we confirmed that WSC contained scopolin and scopoletin (Supplementary Figure S1). To prepare WSC extract, 500 mg of the dried WS calluses were dissolved in 1 mL of DMSO and extracted by sonication for 1 h, and the supernatant was used in further studies after centrifugation at 12,000 rpm for 5 min.

### 2.2. Antioxidant Effect and Viability of W. subsessilis Callus Extract in RAW264.7 Cells

**Figure 2:**
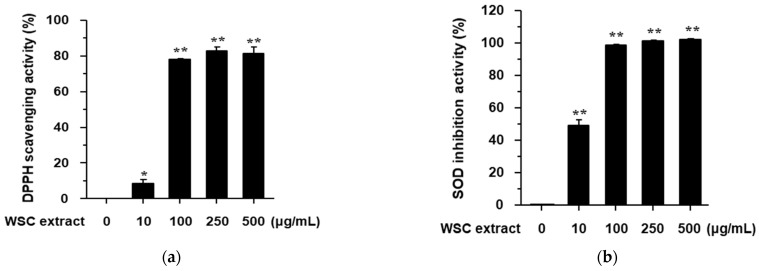
Antioxidant effects of WSC extract: (**a**) The DPPH scavenging activity of WSC extract was measured at the indicated concentrations; (**b**) The SOD inhibition activity of WSC extract was measured at the indicated concentrations. Values are expressed as the mean ± standard deviation of three independent experiments. *, *p* < 0.05; **, *p* < 0.005 versus the control.

A previous study reported that the flower extract of WS has antioxidant and anti-inflammatory effects [4]. However, the antioxidant and anti-inflammatory effects of WSC remain unknown. We found that the DPPH (2,2-diphenyl-1-picryl-hydrazyl-hydrate) radical scavenging and SOD (superoxide dismutase) inhibition activities of WSC extract occurred in a dose-dependent manner (Figure 2). The half-maximal inhibitory concentrations of WS extract were 66 ± 2.56 μg/mL in the DPPH assay and 20.0 ± 3.33 μg/mL in the SOD assay. These findings indicate that the WSC extract has significant antioxidant activity.

### 2.3. Anti-Inflammatory Effect of the WSC Extract on Lipopolysaccharide-Stimulated RAW264.7 Cells

**Figure 3:**
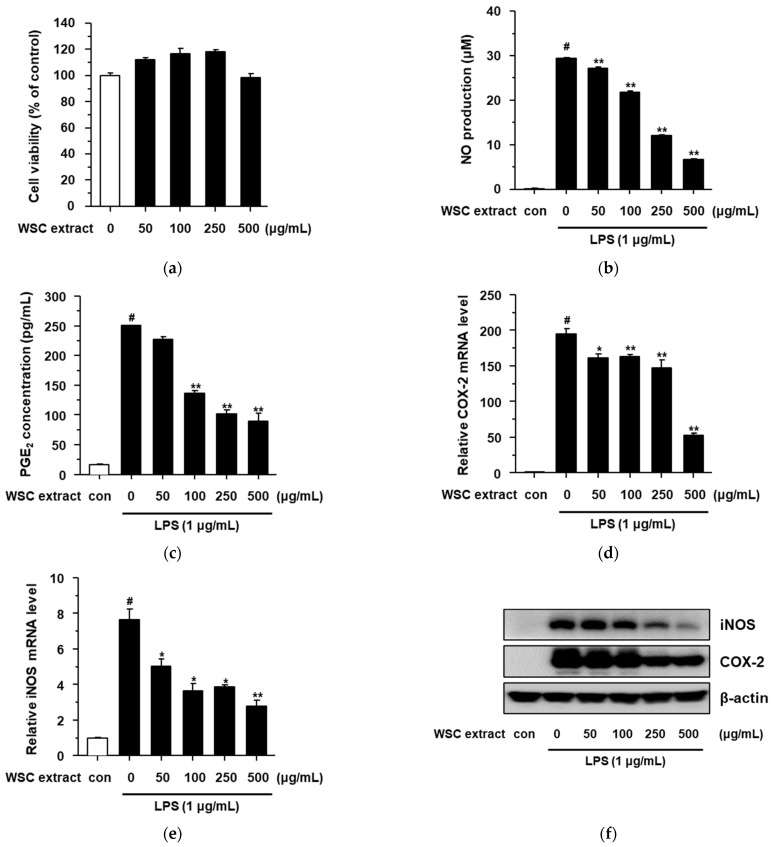
Anti-inflammatory effects of the WSC extract on LPS-stimulated RAW264.7 cells: (**a**) Cytotoxicity of the WSC extract was measured via dimethylthiazol-diphenyltetrazolium bromide assay; (**b**) Griess reaction assay was performed to determine NO production; (**c**) PGE_2_ concentration was determined using an ELISA kit; (**d**,**e**) iNOS and COX-2 mRNA levels were analyzed by quantitative real-time PCR; (**f**) iNOS and COX-2 protein levels were determined by western blotting assay. Values are expressed as the mean ± standard deviation of three independent experiments; #, *p* < 0.005 versus control; *, *p* < 0.05; **, *p* < 0.005 versus LPS alone.

To investigate the anti-inflammatory effect of the WSC extract, the cytotoxicity of the WSC extract was first determined after treating various concentrations (50, 100, 250, and 500 μg/mL) of the WSC extract for 24 h. As shown in Supplementary Figure S2, we also determined the cytotoxicity of the LPS in RAW264.7 cells because LPS can affect cell viability [16]. The WSC extract and the LPS did not show significant levels of cytotoxicity in RAW264.7 cells (Figure 3a and Figure S2). Nitric oxide (NO) and prostaglandin E2 (PGE_2_) are important biomarkers in the inflammatory response [17,18,19]. Therefore, to assess the anti-inflammatory effect of the WSC extract on lipopolysaccharide (LPS)-stimulated RAW264.7 cells, we measured its inhibitory effect on LPS-induced NO and PGE_2_ production. The WSC extract showed an inhibitory effect on LPS-induced NO production under noncytotoxic conditions (Figure 3b). To confirm the anti-inflammatory effect of the WSC extract, the inhibition of PGE_2_ production was investigated in LPS-induced RAW264.7 cells using an enzyme-linked immunosorbent assay (ELISA) kit. The WSC extract reduced the levels of PGE_2_ in LPS-treated RAW264.7 cells in a dose-dependent manner (Figure 3c). These anti-inflammatory effects, such as the inhibitory effects of NO and PGE_2_ production, are known to be regulated by inducible NO synthase (iNOS) and cyclooxygenase (COX)-2 [17,18,19]. Therefore, we analyzed the mRNA and protein levels of iNOS and COX-2. The mRNA levels of iNOS and COX-2 increased with LPS treatment and their upregulated levels decreased after treatment of WSC extract in a dose-dependent manner (Figure 3d,e). We also measured the protein levels of iNOS and COX-2 via western blotting assay (Figure 3f). These findings suggested that WSC extract inhibits NO and PGE_2_ production by suppressing the mRNA and protein levels of iNOS and COX-2.

### 2.4. Effects of the WSC Extract on LPS-Stimulated Pro-Inflammatory Cytokines in RAW264.7 Cells

**Figure 4:**
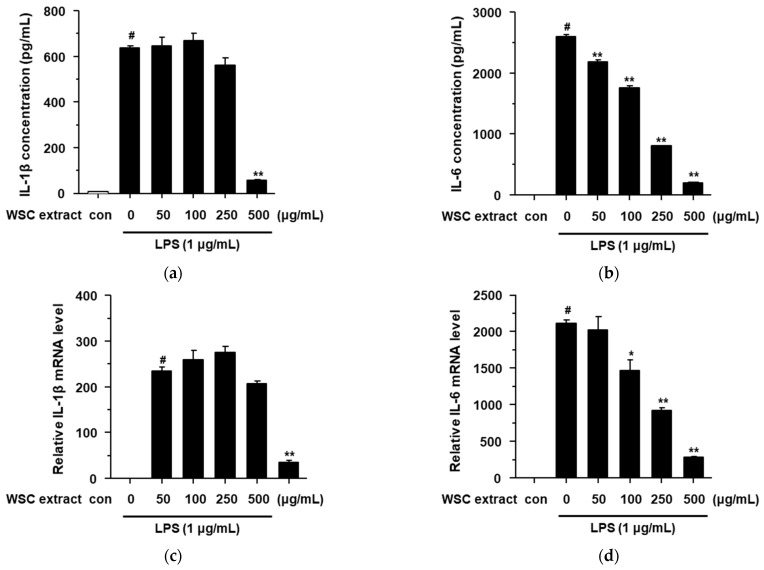
Effects of the WSC extract on LPS-stimulated pro-inflammatory cytokines in RAW264.7 cells. The cells were pretreated with the indicated concentrations of the WSC extract for 3 h and then treated with 1 µg/mL of LPS for 24 h: (**a**,**b**) Protein levels of IL-1β and IL-6 were determined by ELISA; (**c**,**d**) IL-1β and IL-6 mRNA levels were measured by quantitative real-time PCR. Values are expressed as the mean ± standard deviation of three independent experiments; #, *p* < 0.05 versus control; *, *p* < 0.05; **, *p* < 0.005 versus LPS alone.

LPS is a well-known stimulant of inflammatory response, in addition to the pro-inflammatory mediators and cytokines in macrophage cells [20,21,22]. We showed that the WSC extract exhibits anti-inflammatory effects by suppressing pro-inflammatory mediators such as iNOS and COX-2 (Figure 3). Therefore, we evaluated the anti-inflammatory effect of the WSC extract on cytokines such as IL-1β and IL-6. LPS induced the secretion of cytokines (IL-1β and IL-6) in RAW264.7 cells, and these effects were significantly suppressed by the WSC extract in a dose-dependent manner (Figure 4a,b). Next, we determined the mRNA levels of IL-1β and IL-6 in LPS-treated RAW264.7 cells. LPS-induced IL-1β and IL-6 mRNA levels were significantly suppressed by the WSC extract in a dose-dependent manner. These findings suggested that the WSC extract can inhibit the secretion of the cytokines IL-1β and IL-6 by reducing their mRNA levels in LPS-treated RAW264.7 cells.

### 2.5. Effect of the WSC Extract on Mitogen-Activated Protein Kinase Signaling and Nuclear Translocation of NF-κB in LPS-Treated RAW264.7 Cells

**Figure 5:**
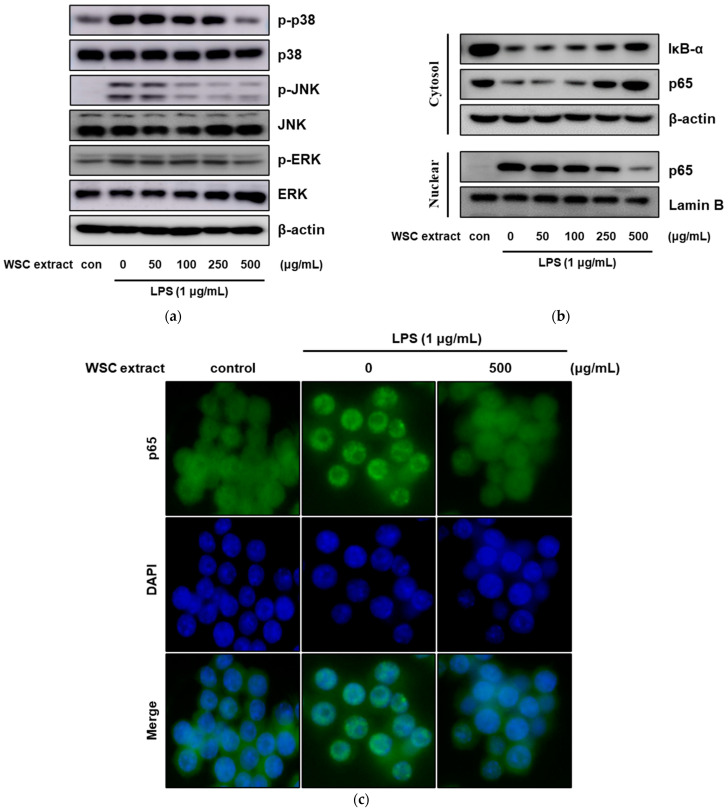
Effect of the WSC extract on MAPK signaling and nuclear translocation of NF-κB in LPS-treated RAW264.7 cells. The cells were pretreated with the indicated concentrations of the WSC extract for 3 h and then treated with 1 µg/mL of LPS for 30 min: (**a**) The protein levels of total and phosphorylated p38, JNK and ERK were determined via western blotting assays; (**b**) The protein levels of IκB-α and NF-κB p65 in cytosolic and nuclear fractions were determined via western blotting assays; (**c**) Localizations of nuclei and NF-κB p65 were visualized following immunofluorescence staining.

It has been reported that LPS stimulates the inflammatory response by activating multiple signaling pathways [23,24]. LPS activates the mitogen-activated protein kinase (MAPK) and nuclear factor-kappa B (NF-κB) pathways by binding to the toll-like receptor 4, eventually contributing to the inflammatory response [25,26]. Therefore, we first investigated whether the anti-inflammatory effects of WSC were regulated via the MAPK signaling pathway. The expression levels of phosphorylated p38, JNK and ERK increased after treatment with LPS, while its effects were significantly decreased by treatment with the WSC extract (Figure 5a). The inactive NF-κB complex induces activation by triggering IκB-α degradation by IκB-α kinase. The activated NF-κB is translocated from the cytoplasm to the nucleus, and subsequently increases the transcription of inflammatory proteins. Therefore, to assess the effect of the WSC extract on the LPS-induced NF-κB signaling pathway, the expressions of IκB-α and NF-κB p65 were investigated via western blotting assay. The protein levels of IκB-α decreased after treatment with LPS and subsequently, NF-κB p65 was translocated from the cytoplasm to the nucleus; these effects were significantly decreased after treatment with the WSC extract (Figure 5b). Furthermore, the translation of NF-κB p65 via LPS was confirmed by immunofluorescence staining (Figure 5c). These results suggested that the WSC extract can inhibit the LPS-induced inflammatory response by suppressing both the MAPK signaling pathway and LPS-induced translocation of NF-κB.

### 2.6. Antibacterial and Anti-Inflammatory Effects of the WSC Extract

**Figure 6:**
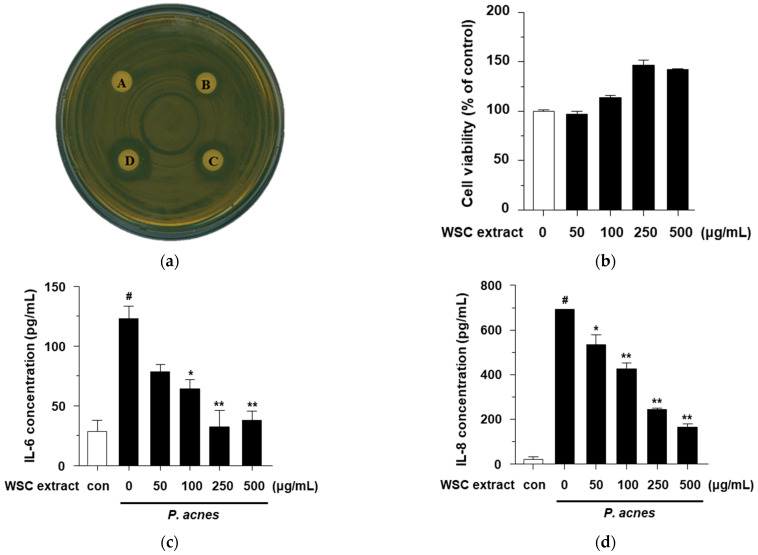
Antibacterial and anti-inflammatory effects of the WSC extract: (**a**) Antibacterial effects of the WSC extract measured by agar disk diffusion assay (**A**, DMSO; **B**, 1 mg/disk of WSC extract; **C**, 2 mg/disk of WSC extract; **D**, 3 mg/disc of WSC extract); (**b**) Cytotoxicity of the WSC extract was measured in HaCaT cells by dimethylthiazol-diphenyltetrazolium bromide assay; (**c**,**d**) Secretion levels of IL-6 and IL-8 were determined using an ELISA kit. Values are expressed as the mean ± standard deviation of three independent experiments; #, *p* < 0.05 versus control; *, *p* < 0.05; **, *p* < 0.005 versus LPS alone.

Even though the antibacterial and anti-inflammatory effects of the WS extract on *Propionibacterium acnes*-induced inflammation in HaCaT cells are unknown, WS is known to have chemical constituents with antibacterial properties [9]. Therefore, the antibacterial effects of the WSC extract against *P. acnes* were investigated. To determine the antibacterial activity of the WSC extract, an agar disk diffusion assay, official method for routine antimicrobial susceptibility testing, was performed. Even though not all bacteria can be tested accurately by this method, antibacterial activity against *P. acnes* has been standardized and used in many studies [27]. The antibacterial activity is evaluated by inhibiting the germination and growth of microorganisms and measuring the diameter of the inhibited growth zone. As shown in Figure 6a, at concentrations of 1 mg/disk, 2 mg/disk and 3 mg/disk, the WSC extract resulted in clear zones with diameters of 1.2 mm, 1.5 mm and 2.0 mm, respectively, and dimethyl sulfoxide (DMSO) used as a negative control. This finding indicates that the WSC extract could inhibit the growth of *P. acnes*.

*P. acnes*, linked to acne vulgaris and skin disease, stimulates the proinflammatory cytokines, including IL-6 and IL-8, in keratinocytes [28,29]. Therefore, we investigated the anti-inflammatory effects of the WSC extract by measuring the inflammatory cytokines in *P. acnes*-treated HaCaT cells. We first determined that the WSC extract did not show cytotoxic effect up to a concentration of 500 μg/mL (Figure 6b). Next, we evaluated the anti-inflammatory effect of the WSC extract on cytokines such as IL-6 and IL-8. P. acnes induced the secretion of cytokines (IL-6 and IL-8) in HaCaT cells, and these effects were significantly suppressed by the WSC extract in a dose-dependence manner (Figure 6c,d). These results suggest that the WSC extract possesses an antibacterial activity against *P. acnes* and exhibits anti-inflammatory activity by inhibiting the production of the pro-inflammatory cytokines IL-6 and IL-8 in *P. acnes*-treated HaCaT cells.

## 3. Materials and Methods

### 3.1. Callus Induction and Proliferation of W. subsessilis

Whole plants of WS (Nakai) L.H. Bailey were collected from Jeongeup-si, Jeollabuk-do province in 2017 (Figure 1a). Whole plants were surface-sterilized in 70% (*v*/*v*) ethanol for 0.5 min followed by 1% sodium hypochlorite (NaOCl) solution for 20 min with occasional agitation. Plants were rinsed over three times with sterile distilled water to remove the remaining NaOCl solution thoroughly. After washing, the remaining moisture was removed with sterilized filter paper (Advantec No. 2, 70 mm). The plants were dissected into three segments: the leaf, petiole and stem. To induce callus formation, stem explants were cut into small segments (approximately 5 mm in length). Stem explants were placed onto half-strength Murashige and Skoog [30] medium supplemented with 1 mg/L of benzyl aminopurine, 0.3 mg/L of 2,4-dichlorophenoxyacetic acid, 0.4 mg/L of thiamine-HCl, 0.6% myo-inositol, 3% (*w*/*v*) sucrose and 0.4% (*w*/*v*) Gelrite (1/2MS1B03D). Each treatment consisted of 10 explants with three replicates. Unless otherwise indicated, all cultures were maintained at 25 °C in the dark (24 h). All reagents related to plant tissue culture were purchased from Duchefa Biochemie (Haarlem, The Netherlands) and plant growth regulators were purchased from Sigma–Aldrich (St. Louis, MO, USA).

### 3.2. HPLC Apparatus and Conditions

The HPLC analysis for comparison of the calluses and leaves of WS in vitro was performed using an Agilent 1200 series HPLC system (Agilent Technologies, Palo Alto, CA, USA), equipped with a diode array detector, binary gradient pump, autosampler and vacuum degasser. The indicated compounds were eluted using a Capcell Pak MGII ODS column (C18, 4.6 mm I.D. × 150 mm, 3 μm particle size) at 35 °C with a flow rate of 0.35 mL/min. The injection volume was 15 µL with needle wash. The mobile phase comprised a mixture of aqueous formic acid (0.1%, *v*/*v*) and 0.5% acetonitrile. Scopolin and scopoletin (Sigma–Aldrich) as markers, were eluted under gradient conditions. Data were collected and integrated using Agilent Chemstation B.04.01 software. The standard solutions of scopolin and scopoletin were prepared using 70% aqueous methanol (*v*/*v*). The in vitro callus and leaves of WS were extracted with the same solvent used in the standard solution and compared using HPLC analysis.

### 3.3. Preparation of the WSC Extract

After four weeks of incubation, rapidly growing calluses derived from stem explants of WS were transferred to fresh 1/2MS1B03D medium and further incubated in the dark at 25 °C (Figure 1b). The calluses of WS were maintained by subcultures at four-week intervals. These calluses were collected carefully, freeze-dried and then ground into a fine powder. These calluses were carefully transferred to the tube (Falcon, 50 mL) without agar fragments. Collected calluses were freeze-dried and pulverized into fine powder using a pestle and mortar. The fine powder of WS was stored at −70 °C prior to analysis. Crude whole-cell extracts were prepared from each callus as follows: 500 mg sample of each callus powder was mixed with 1 mL of DMSO. After sonication for 1 h, the mixtures were centrifuged at 12,000 rpm for 5 min. In addition, the supernatants were transferred to fresh tube. These crude whole-cell extracts (WSC extract) used in this study.

### 3.4. Cell Culture and Viability

To evaluate the anti-inflammatory effects, we used The RAW264.7 mouse macrophage cells (The Korean Cell Line Bank, Seoul, Korea) and the human keratinocyte HaCaT cells (Cell Lines Service GmBH, Eppelheim, Germany). The cells were cultured in Dulbecco’s Modified Eagle Medium (WELGENE Inc., Seoul, Korea) supplemented with 10% fetal bovine serum (Hyclone Laboratories Inc., Logan, UT, USA) at 37 °C under a humidified atmosphere comprising 5% carbon dioxide. The cytotoxic effects of WSC toward RAW 264.7 cells and HaCaT cells were assessed via dimethylthiazol-diphenyltetrazolium bromide (MTT) assay. The cells were seeded into 96-well plates at a density of 5 × 10^3^ cells/well in an incubator overnight. WSC was dissolved in DMSO (at concentrations not exceeding 0.1% in each assay). After incubating overnight, the cells were treated with various concentrations (0–500 μg/mL) of WSC for 24 h. After adding 5 mg/mL of MTT reagent to each well, the cells were incubated for 4 h. After removal of the supernatant, the cells were solubilized with DMSO, and the absorbance was measured at 570 nm using a microplate reader (Thermo Fisher Scientific, Waltham, MA, USA).

### 3.5. Antibacterial Activity

Antibacterial activity was evaluated using an agar disk diffusion method [26]. In brief, *P. acnes* (The American Type Culture Collection, Manassas, VA, USA) was cultured with brain heart infusion (BHI) (BD Biosciences, San Jose, CA, USA) under anaerobic conditions at 37 °C. To investigate the antibacterial activity of the WSC, filter paper disks of 8 mm diameter were treated with disks containing 1–3 mg/mL of WSC or DMSO and placed on the surface of BHI agar plates. The disks were then incubated for 48 h at 37 °C. Clear zones of growth inhibition around the disks were measured in mm.

### 3.6. DPPH Assay

The DPPH scavenging activity of WSC was determined using a previously described method [31]. In brief, 100 µL of WSC samples (0–500 µg/mL) was added to 100 µL of 0.1 mM DPPH ethanol solution. After 30 min incubation in the dark, absorbance was measured at 517nm using a microplate reader (Thermo Fisher Scientific). The DPPH scavenging ability was calculated using the following formula:

DPPH radical scavenging activity (%) = 1 – (Absorbance of control – Absorbance of sample)/Absorbance of control × 100.

### 3.7. SOD Assay

The SOD activity of WSC was assessed using a SOD assay kit (Sigma–Aldrich). In brief, 20 μL of WSC samples (0–500 μg/mL) was added to 220 μL of the reaction mixture and incubated at 37 °C for 20 min. After 20 min incubation in the dark, absorbance was measured at 450 nm using a microplate reader (Thermo Fisher Scientific). The SOD activity was calculated to follow manufacturer’s instructions.

### 3.8. Nitric Oxide Production Assay

RAW264.7 cells were seeded at a density of 5 × 10^4^ cells/well in 24-well plates. These cells were pretreated with various concentrations (0–500 μg/mL) of WSC for 3 h prior to LPS (1 μg/mL) stimulation for 24 h. The supernatant was collected from each well for NO determination. The concentrations of NO in the supernatants were measured using Griess reagent (Promega Corporation, Madison, WI, USA) according to the manufacturer’s protocols.

### 3.9. Determination of Inflammatory Cytokine and PGE_2_ Level

RAW264.7 cells were seeded at a density of 5 × 10^4^ cells/well in 24-well plates. These cells were pretreated with various concentrations (0–500 μg/mL) of WSC for 3 h prior to stimulation with 1 μg/mL of LPS for 24 h. The supernatant was collected from each well for IL-1β (#MLB00C), IL-6 (#M6000B) and PGE_2_ (#KFE004B) determination. The levels of expression were measured using an ELISA kit (R&D Systems, Minneapolis, MN, USA) according to the manufacturer’s protocols. HaCaT cells were seeded at a density of 5 × 10^4^ cells/well in 24-well plates. These cells were pretreated with various concentrations (0–500 μg/mL) of WSC for 4 h prior to stimulation with heat-killed *P. acnes* for 18 h. The supernatant was collected from each well for IL-6 (#555220) and IL-8 (#555224) determination. The levels of expression were measured using an ELISA kit (BD Biosciences) according to the manufacturer’s protocols.

### 3.10. Quantitative Real-Time Polymerase Chain Reaction (qRT-PCR)

Total RNA was isolated from the cells using TRI Reagent (Molecular Research Center, Cincinnati, OH, USA) according to the manufacturer’s manuals. Total RNA (1 μg) was reverse-transcribed using a 2× cDNA synthesis premix kit (BioFACT, Seoul, Korea). Real-time reverse transcription PCR was performed using SYBR Green qPCR Master Mix (BioFACT) and a real-time PCR system (Bio-Rad Laboratories, Hercules, CA, USA). The primer sequences utilized are shown in Table 1. The relative amounts of mRNA were calculated based on the cycle threshold values using β-actin as a control. All experiments were performed in triplicate and the values were averaged.

### 3.11. Western Blot Analyses

Cells were harvested with phosphate-buffered saline (PBS, Hyclone), lysed using radioimmunoprecipitation assay buffer (RIPA, Thermo Fisher Scientific) and separated using a nuclear extract kit (Thermo Fisher Scientific). Protein concentrations were determined using a bicinchoninic acid protein assay kit (Bio-Rad Laboratories). Equal amounts of protein (50 μg) were separated using 10% sodium dodecyl sulfate-polyacrylamide gel electrophoresis (SDS-PAGE) and then transferred to polyvinylidene difluoride (PVDF) membranes (Merck KGaA, Darmstadt, Germany). Membranes were blocked with 5% bovine serum albumin (BSA)/TBST (tris-buffered saline with 0.1% Tween 20) for 2 h at room temperature. Afterward, the mixtures were incubated at 4 °C overnight with the following primary antibodies: anti-iNOS (#13120), anti-COX-2 (#12282), anti-p38 (#9212), anti-phospho p38 (#9211), anti-phospho ERK (#9101), anti-phospho JNK (#4668), anti-p65 (#8242), anti-phospo p65 (#3033), anti-IκB-α (#4812) and anti-β-actin (#3700) obtained from Cell Signaling Technology (Danvers, MA USA), and anti-ERK (sc-514302), anti-JNK (sc-7345) and Lamin B (sc-374015) obtained from Santa Cruz Biotechnology Inc. (Santa Cruz, CA, USA). Blots were washed thrice for 15 min with TBST and incubated with secondary antibodies for 30 min. Protein bands were detected using an enhanced chemiluminescence kit and Amersham Imaging System (GE Healthcare, Buckinghamshire, UK).

### 3.12. Immunofluorescence Staining

RAW264.7 cells were plated on cover glasses and pretreated with various concentrations (0–500 µg/mL) of WSC for 3 h prior to LPS stimulation for 30 min. Afterward, the cells were fixed with 4% paraformaldehyde in PBS for 15 min and then permeabilized with 0.2% Triton X-100 for 15 min. The fixed cells were blocked in 5% BSA for 1 h and then incubated with the primary antibodies (1:1000) at 4 °C overnight. Then, the cells were incubated with the fluorescein isothiocyanate-conjugated secondary antibody for 1 h at room temperature. For nuclear staining, 4′,6-diamidino-2-phenylindole solution (10 mg/mL) was added at room temperature for 10 min. Images were analyzed using a fluorescence microscope (Olympus, Tokyo, Japan).

### 3.13. Statistical Analysis

All results are presented as the mean ± standard deviation. The statistical analyses were performed using GraphPad Prism version 5.0 (GraphPad Software, Inc., La Jolla, CA, USA) with Tukey’s posthoc tests. For all experiments, analysis items with *p* < 0.05 were considered statistically significant.

## 4. Conclusions

In this study, to evaluate whether the WSC extract retains the pharmacological activities, such as antioxidant, antibacterial and anti-inflammatory, of the parent plant, we established an in vitro proliferation system for the callus of WS and evaluated. We showed that WSC have antioxidant, antibacterial and anti-inflammatory activities against LPS-induced and *P. acnes*-induced inflammation. The WSC extract elicited these activities by suppressing the release of pro-cytokines via inhibition of both MAPK and NF-κB signaling pathways. These findings suggest that the WSC extract might have applications in both cosmetic and biotechnology industries to treat inflammatory disorders.

## Figures and Tables

**Table 1:** Primer sequences for real-time PCR.

Gene		Sequence (5′ to 3′)
*COX-2*	Forward	CTCTACAACAACTCCATCCT
(mouse)	Reverse	ATTCTGCAGCCATTTCCTTC
*iNOS*	Forward	GTCCTACACCACACCAAACT
(mouse)	Reverse	AATCTCTGCCTATCCGTCTC
*IL-1β*	Forward	ACCTGTGTCTTTCCCGTGG
(mouse)	Reverse	TCATCTCGGAGCCTGTAGTG
*IL-6*	Forward	TGTCTATACCACTTCACAAGTCGGAG
(mouse)	Reverse	GCACAACTCTTTTCTCATTTCCA
*β* *-actin*	Forward	CGGTTCCGATGCCCTGAGGCTCTT
(human)	Reverse	CGTCACACTTCATGATGGAATTGA

## Data Availability

Not applicable.

## Supplementary Materials

The following are available online at https://www.mdpi.com/article/10.3390/plants10081635/s1, Figure S1: Chemical structure of standard compounds., Figure S2: Cytotoxicity of the WSC extract and LPS.
