# Supplementary material for: Anti-Inflammatory Effects of Weigela subsessilis Callus Extract via Suppression of MAPK and NF-κB Signaling
Source: Plants (Basel). 2021 Aug 9;10(8):1635. doi: 10.3390/plants10081635 (PMC8400516; doi:10.3390/plants10081635)
Supplement: Supplementary file 1 [file plants-10-01635-s001.zip › plants-1326820-supplementary.pdf]

Anti-inflammatory effects of *Weigela subsessilis* callus extract via suppression of MAPK and NF-κB signaling

Hyeon-Ji Lim [1], Eun Yee Jie [2], In-Sun Park [1], Sang-Jun Kim [1], Woo Seok Ahn [2], Seung-Il Jeong [1], Suk Weon Kim [2,*] and Chan-Hun Jung [1,*]

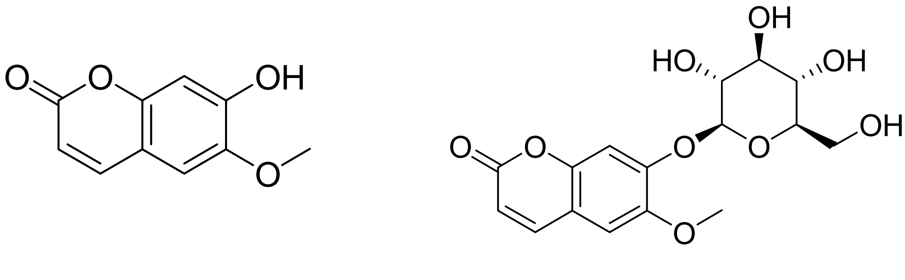

(A) **(B)**

Figure S1. Chemical structure of standard compounds. (A) Scopolin. (B) Scopeletin.

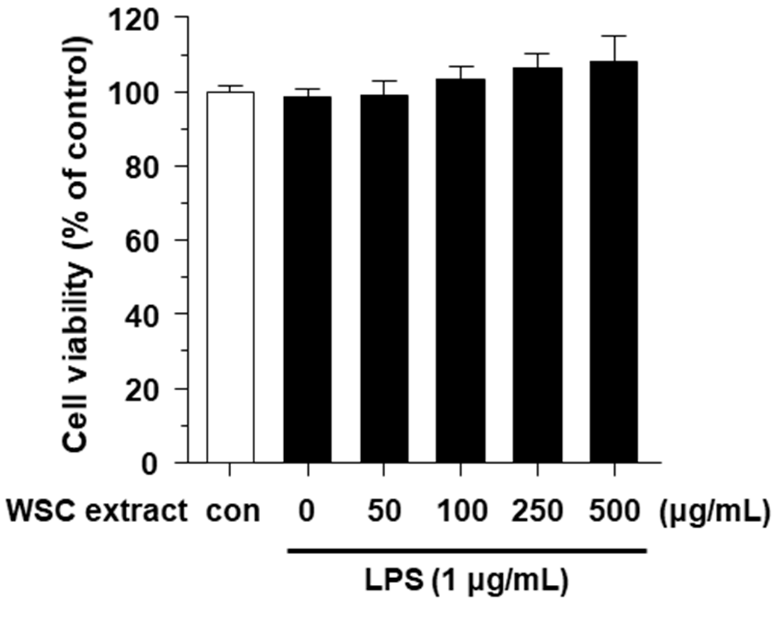

Figure S2. Cytotoxicity of the WSC extract and LPS. Cell viability was measured after treatment of both 1 µg of LPS and indicated concentration of WSC extract in RAW264.7 cells by dimethylthiazol-diphenyltetrazolium bromide assay. Values are expressed as mean ± SD of three independent experiments.
